# Characterization of Two α-l-Arabinofuranosidases from *Acetivibrio mesophilus* and Their Synergistic Effect in Degradation of Arabinose-Containing Substrates

**DOI:** 10.3390/microorganisms9071467

**Published:** 2021-07-08

**Authors:** Yajing Liu, Sonja Vanderhaeghen, Werner Feiler, Angel Angelov, Melanie Baudrexl, Vladimir Zverlov, Wolfgang Liebl

**Affiliations:** Chair of Microbiology, TUM School of Life Sciences, Technical University of Munich, Emil-Ramann-Straβe 4, D-85354 Freising, Germany; yajing.liu@tum.de (Y.L.); s.vanderhaeghen@tum.de (S.V.); wernerfeiler@gmx.de (W.F.); angelov.tum@gmail.com (A.A.); melanie.baudrexl@tum.de (M.B.); vladimir.zverlov@tum.de (V.Z.)

**Keywords:** α-l-arabinofuranosidase, substrate specificity, bifunction, synergism, *Acetivibrio mesophilus*

## Abstract

Arabinofuranosidases are important accessory enzymes involved in the degradation of arabinose-containing poly- and oligosaccharides. Two arabinofuranosidases from the recently described novel anaerobic cellulolytic bacterium *Acetivibrio mesophilus*, designated *Am*Araf51 and *Am*Araf43, were heterologously expressed in *Escherichia coli* and biochemically characterized. *Am*Araf51 not only removed arabinose moieties at O-3, O-2 and terminal O-5 positions of arabinose-containing oligosaccharides, but also exhibited exo-β-xylosidase side activity. In comparison, *Am*Araf43 preferably cleaved 1,3-linkages from arabinosyl disubstitutions. *Am*Araf51 and *Am*Araf43 demonstrated maximum activity at 70 °C and 57 °C, respectively. Judging from the genetic context and substrate specificity, *Am*Araf51 may decompose internalized arabino/xylo-oligosaccharides. The embedding of the *Am*Araf43 gene between genes for several putative xylanolytic enzymes, along with its enzymatic properties suggests that *Am*Araf43 cleaves arabinose decorations from heteroxylans extracellularly. The enzymes revealed completely converse activity profiles towards arabinan/arabinoxylan: *Am*Araf51 displayed strong activity on arabinan, while *Am*Araf43 prefers arabinoxylan. *Am*Araf51 dramatically stimulated the saccharification level of wheat arabinoxylan (WAX-RS) and sugar beet arabinan when administered along with xylanase M_Xyn10 or arabinanase *Pp*Abn43, respectively. For WAX-RS degradation, the yield of arabinose and xylose was boosted 13.77-fold and 4.96-fold, respectively. The bifunctional activity, thermostability and high catalytic efficiency make *Am*Araf51 an interesting candidate for industrial applications.

## 1. Introduction

With future limitations in the availability of fossil resources looming and increasing environmental concerns, the use of lignocellulosic biomass for the production of bio-based fuels and chemicals has increasingly attracted global attention. However, due to the recalcitrant structure of lignocellulose, its enzymatic deconstruction is still a major scientific and practical challenge and remains a topic demanding further research efforts [1]. Lignocellulose is mainly comprised of three primary polymers: cellulose (40.6–51.2%), hemicelluloses (28.5–37.2%) and a lower fraction of lignin (13.6–28.1%) [2], which form an intricate matrix.

Various plant cell wall polymers contain arabinose, which is the second most abundant pentose in nature [3]. Arabinose-containing polysaccharides such as arabinoxylan (AX), arabinan and arabinogalactan (AG) are found as major components of hemicelluloses and pectic substances, respectively. AX has a linear backbone of β-1,4-linked d-xylopyranosyl units, which are randomly single or double substituted at the O-3 and/or O-2 position by l-arabinose moieties [4]. Some of the arabinose residues are linked to ferulic acid by ester bonds and the formation of ferulate dimers creates arabinoxylan-arabinoxylan cross-links [5]. Arabinans and arabinogalactans are constituents of pectic substances [6]. Arabinan forms side-chains in the rhamnogalacturonan I or occurs as a free polysaccharide. It consists of a linear α-1,5-linked l-arabinofuranosyl polymer as the backbone, which can be decorated with arabinofuranosyl residue at O-3 and/or O-2 position, such as in sugar beet arabinan (SBA) [7], without its side chains this polysaccharide is called debranched arabinan (DA). AG consists of β-1,6-glycosidically linked d-galactopyranose residues as the backbone, which is modified by side chains of α-arabinose, β-galactose and 4-*O*-methylglucuronic acid [8]. All these arabinose-containing polymers of hemicellulose and pectic substances play an important role in the crosslinking within the plant cell wall structure and thus increase the complexity of plant cell wall architecture. 

For complete deconstruction of arabinose-containing polysaccharides, microorganisms produce various enzymes with different substrate and cleavage specificities, such as endo-β-1,4-xylanase, β-1,4-xylosidase, endoarabinanase, α-l-arabinofuranosidase or β-1,3-galactosidase [9,10]. Glycoside hydrolase family 43 (GH43) encompasses various structurally related enzymes with an inverting hydrolysis mechanism involved in the degradation of arabinose-containing carbohydrates [11]. For example, endoarabinanase is capable of hydrolyzing the α-1,5-linked l-arabinofuranosyl backbone of arabinans to produce arabino-oligosaccharides, exo-α-1,5-arabinanases are active on the non-reducing end of arabinan chain releasing arabinobiose or arabinotriose [12,13], whereas α-l-arabinofuranosidase cleaves off side chain decorations in arabino-oligosaccharides or arabinose-containing polysaccharides, e.g., with xylan, galactan or arabinan backbones, thus liberating l-arabinose [14]. However, arabinofuranosyl di-substitutions such as found on xylose residues of certain xylans withstand cleavage by most known arabinofuranosidases [15]. Besides GH43, arabinofuranosidases are also found as members of GH3, GH51, GH54 and GH62 [11]. According to their cleavage specificities in arabinoxylan degradation, arabinofuranosidases are grouped into different types [16]: As the most common type of arabinofuranosidases, AXHs-m remove arabinose moieties from 1,2- or 1,3-monosubstituted main-chain xylose residues, which can be found in most of the mentioned GH families [10,17], while AXHs-d cleave off either α-1,2- or α-1,3-linked arabinofuranosyl side chains from double-substituted main-chain xylose residues, additional numbers can be used to describe a more specific cleavage mode, such as AXH-d3 refers to the enzymes that only cleave off 1,3-linked arabinofuranosyl residue from double substituted Xylp motifs [18,19,20] and AXHs-m,d finally remove arabinofuranosyl substitutions from monosubstituted and double-substituted xylose residues [18,21]. Some α-l-arabinofuranosidases are also able to cleave off terminal α-l-arabinofuranosyl residues from decorated or linear arabinan [21].

Strain N2K1 is a novel cellulolytic organism, recently described as *Hungateiclostridium mesophilum* sp. nov. and shortly reclassified as *Acetivibrio mesophilus*, which was isolated from a mesophilically operated biogas plant fed with maize silage [22,23]. The physiological characterization of this organism revealed its high potential for degrading recalcitrant substrates from plant biomass (wheat arabinoxylan, oat spelt xylan, sugar beet pulp and cellulose), so this organism can be regarded as an attractive source of enzymes for biotechnological applications. The 4.04 Mbp genome assembly of strain N2K1^T^ was annotated to contain a wide variety of glycoside hydrolase genes for the depolymerization of the major polysaccharides in plant cell walls. However, while gene annotation mostly considers sequence similarity or secondary structures, the true enzymatic function of the putative proteins often cannot be predicted accurately, even if high sequence similarity to other enzymes is evident [24].

In the genome sequence of *A. mesophilus* N2K1, we found an approximately 26 kbp large gene cluster of colinearly arranged genes for glycoside hydrolases, transport proteins and regulatory proteins putatively involved in hemicellulose and pectin substrate degradation. Here we mainly focus on the enzymes with the potential for degradation of arabinose-containing heteroxylan or pectic arabinan encoded in this gene cluster. Specifically, we chose two arabinofuranosidases belonging to the GH families GH43 and GH51 and designated *Am*Araf43 and *Am*Araf51, respectively, for characterization and comparison. Furthermore, we also explored the synergistic action modes of these enzymes on SBA and AX degradation by the combination with endoactive polysaccharide hydrolases.

## 2. Materials and Methods

### 2.1. Bacterial Strains and Plasmids

*Acetivibrio mesophilus* (basonym. *Hungateiclostridium mesophilum*) strain N2K1^T^ is a novel anaerobic, mesophilic and cellulolytic bacterium isolated from a biogas fermenter, as recently described by Rettenmaier et al. [22,23]. Genomic DNA from this strain (NCBI Reference Sequence ID: 85920) served as a template for gene amplification. The *E. coli* strain XL1-Blue (Stratagene, La Jolla, CA, USA) was used for general cloning purposes, propagation of recombinant plasmids and cloning. The *E. coli* strain ArcticExpress (DE3) was used as a host for protein production by gene expression from pET24c vectors. The bacteria were cultivated in lysogeny broth (LB) or grown on LB agar plates supplemented with the appropriate antibiotics. Kanamycin was used at 50 μg mL^−1^ and gentamycin at 20 μg mL^−1^.

### 2.2. Gene Cloning and Protein Purification

Genomic DNA of strain N2K1 served as a template for amplification of the genes with GenBank (NCBI) numbers RXE58498.1 (*Am*Araf51) and RXE58512.1 (*Am*Araf43), which encode a putative α-1,5-l-arabinofuranosidase (*Am*Araf51) and a putative endo-1,4-β-xylanase (*Am*Araf43), respectively. The primers used in PCR reactions include *Am*Araf51_f, ATGCATCATCACCATCACCATAAAAAAGCCAGAATGACC, and *Am*Araf51_r, CAGTGGTGGTGGTGGTGGTGCGTTATTTCCCCAGTCGAATTAC, for the gene RXE58498.1. The generated PCR product then served as a template for a second PCR reaction with the same reverse primer but a different forward primer, ACTTTAAGAAGGAGATATACAATGCATCATCACCATCACCAT, to introduce six in-frame histidine codons at the 5′ end of the target ORF. The primers for amplification of the target gene RXE58512.1 encoding *Am*Araf43 were *Am*Araf43_f, CTTTAAGAAGGAGATATACATATGTTATTTACAAAAAAAGCC, and *Am*Araf43_r, TCAGTGGTGGTGGTGGTGGTGCGCTTCAATAAAAGTAAACCAATTC, which included the sequence for the in-frame 3′-extension of the target ORF with six histidine codons.

The amplicons were then cloned in *NdeI*/*XhoI* linearized pET24c vector by the Gibson Assembly Master Mix (New England Biolabs). All the recombinant plasmids were subsequently transformed into competent cells of *E. coli* strain XL1 by heat shock transformation. Correct plasmid construction was verified by restriction analysis and DNA sequencing before their introduction into expression host *E. coli* ArcticExpress. An ArcticExpress transformant carrying the recombinant plasmid was inoculated into 5 mL of lysogenic broth (LB) medium supplemented with 50 μg mL^−1^ kanamycin and 20 μg mL^−1^ gentamycin and grown aerobically at 37 °C overnight. The overnight culture was inoculated into 1 L of LB and incubated with shaking (180 rpm) at 30 °C for 6 h before addition of 1 mM isopropyl β-d-1-thiogalactopyranoside (IPTG) and incubation at 13 °C, 250 rpm for 24 h. The cells were harvested and disrupted by sonication with a Hielscher UP200S apparatus (Hielscher Ultrasonics GmbH, Teltow, Germany) for 5 min (50% amplitude, 0.4 s cycle), and the cell-free crude extract was prepared by centrifuging the cell lysate at 13,400× *g*, at 4 °C for 30 min followed by filtration through a 0.45 µm filter. *Am*Araf51 and *Am*Araf43 were purified by immobilized metal affinity chromatography (IMAC) using a nickel column (Machery-Nagel Protino^®^ Ni-TED 2000, Fischer Scientific GmbH, Schwerte, Germany). The elution fraction containing the soluble protein was further purified by using an ÄKTA pure 25L1 FPLC system (GE Healthcare Life Sciences, Amersham, UK) equipped with a HiTrap^TM^ 1 mL QFF column, using a linear gradient from 0 to 1 M NaCl. The size and purity of proteins were analyzed by sodium dodecyl sulfate polyacrylamide gel electrophoresis (SDS-PAGE) followed by pooling the fractions containing pure enzyme. The protein concentration was determined with the Bradford assay with bovine serum albumin (BSA) as a standard.

### 2.3. Enzyme Assays

Arabinoxylan (from wheat flour, insoluble; WAX-I), arabinoxylan (from wheat flour for the reducing sugar assay; WAX-RS), arabinan (from sugar beet pulp; SBA), debranched arabinan (from sugar beet pulp, arabinose:galactose:rhamnose = 71:26:3; DA), linear arabinan (from sugar beet pulp, arabinose: galactose:rhamnose:galacturonic acid = 85.2:7.6:1.5:5.7; LA), arabino-oligosaccharides (AOS), xylo-oligosaccharides (XOS) and arabino-xylo-oligosaccharides (AXOS) were purchased from Megazyme (Wicklow, Ireland), Beechwood xylan were purchased from SERVA Electrophoresis GmbH (Heidelberg, Germany). WAX-I had an Araf:Xylp ratio of 36:51 while still maintaining ferulic acid crosslinks during the process of substrate extraction, while WAX-RS had an Araf:Xylp ratio of 38:62 with diferulate bridges mostly broken due to alkaline treatment during substrate extraction [21,25]. All insoluble substrates used in this study were washed with Milli-Q water followed by centrifugation before starting the enzyme assay. The optimal temperature (at pH 6.0 for both *Am*Araf51and *Am*Araf43) and pH (at 70 °C for *Am*Araf51 or 57 °C for *Am*Araf43) of the enzymes were tested in the pH range between pH 2.0 and 9.0 (pH adjusted at 60 °C) and the temperature range between 25 and 90 °C, respectively, with appropriately diluted enzyme in 25 mM citrate-phosphate buffer (citric acid, Na_2_HPO_4_, 50 mM NaCl) using *p*-nitrophenyl-α-l-arabinofuranoside (*p*NP-AF) (Megazyme) as the substrate at a final concentration of 0.2 mM. The reaction was stopped after 20 min by adding two volumes of 1 M Na_2_CO_3_, followed by a photometrical measurement at 405 nm. The resistance of the enzymes against thermoinactivation was measured by comparing their changes in activity against *p*NP-AF, before and after heat treatment for various time spans (0 h, 30 min, 1 h, 2 h, 4 h, 6 h, 8 h,16 h, 18 h, 24 h and 48 h).

The specific activities of the enzymes on arabinose/xylose-based polysaccharide substrates were measured using the substrates at a final concentration of 5 g L^−1^ in 25 mM citrate-phosphate buffer at their optimal pH and appropriate enzyme concentrations (between 360 nM to 2 µM). The reactions were performed at optimal temperature with 600 rpm shaking on an Eppendorf thermomixer. At specific intervals, aliquots of 200 μL were withdrawn and cooled on ice. For the insoluble substrate, aliquots were centrifuged at 4 °C for 5 min at 13,000 rpm, then the supernatant was transferred to new tubes for further analysis. The reducing ends liberated during the enzymatic hydrolysis reactions were quantified with the 3,5-dinitrosalicylic acid (DNS) reducing sugar method [26]. To this end, 50 µL samples of the enzyme reactions were mixed with 75 µL of DNS reagent, followed by incubation for 10 min at 95 °C in 96 well PCR plates in a thermocycler. The products were then transferred into wells of 96 well microtiter plates and the absorbance were measured at 540 nm. All assays were performed in triplicate. One unit of activity was defined as the amount of enzyme needed to release 1 µmol of l-arabinose equivalent per minute. 

Kinetic parameters were determined on SBA and washed DA for *Am*Araf51 and on WAX-RS for *Am*Araf43 by incubating at 60 °C and at 50 °C, respectively, under shaking at 600 rpm using an Eppendorf thermomixer, 200 µL of reaction mixtures containing 25 mM citrate-phosphate buffer, various concentrations of substrates (45 g L^−1^, 40 g L^−1^, 35 g L^−1^, 30 g L^−1^, 25 g L^−1^, 15 g L^−1^, 10 g L^−1^, 7.5 g L^−1^, 5 g L^−1^, 2.5 g L^−1^ and 1 g L^−1^ for SBA and DA; 25 g L^−1^, 20 g L^−1^, 15 g L^−1^, 10 g L^−1^, 7.5 g L^−1^, 5 g L^−1^, 2.5 g L^−1^ and 1 g L^−1^ for WAX-RS) and different concentrations of the enzyme (final concentration of 30 µg mL^−1^, 20 µg mL^−1^ and 10 µg mL^−1^ for *Am*Araf51 and 70 µg mL^−1^, 50 µg mL^−1^ and 30 µg mL^−1^ for *Am*Araf43), after two hours incubation at 60 °C (*Am*Araf51) or 50 °C (*Am*Araf43), aliquots were cooled on ice and centrifuged at 4 °C for 5 min at 13,000 rpm. Then, 50 µL of supernatant was mixed with 75 µL of DNS reagent in 96 well plates for the DNS assay and absorbance readout at 540 nm as described above. All assays were performed in triplicate.

To explore the enzymatic action mode and preference towards the specific side chains of arabino-/xylo-oligosaccharides, the oligosaccharides, including A3, O-A4B, O-A5B, O-XTR, O-XTE, O-XPE, O-XHE, O-XBI, O-A3X, O-AX3, O-XAXX MIX, O-XA3XX, O-A2X3 and O-XA23XX (the structures of the oligosaccharides are shown in Table S1), were individually incubated in a thermocycler at a final substrate concentration of 0.5 g L^−1^ with 85 nM *Am*Araf51 or 82 nM *Am*Araf43 at pH 6.0, 60 °C or pH 5.0, 40 °C, respectively, for 24 h. For the reactions with arabino-oligosaccharide substrates (A3, O-A4B and O-A5B), 10 µL of the reaction mixtures were collected at specific intervals (5 min, 10 min, 30 min, 1 h, 5 h and 20 h), then the reactions were terminated by incubation at 100 °C for 10 min. The hydrolysis products were identified by high performance anion exchange chromatography with pulsed amperometric detection (HPAEC-PAD) using a Dionex ICS 3000 SP system equipped with a CarboPac PA1 column (4 mm × 250 mm) and a PA1 precolumn (4 mm × 50 mm) as described by Angelov et al. [27]. Analytes were injected and the analysis was performed at 30 °C at a flow rate of 1 mL/min. Separation was achieved in 100 mM sodium hydroxide using an increasing sodium acetate gradient within 45 min. The program was set as follows: 0–10 min: a linear gradient from 0 to 100 mM sodium acetate, 10–30 min: a linear gradient from 100 to 800 mM sodium acetate and 30–45 min: 0 mM sodium acetate. Thin layer chromatography (TLC) was also performed for the qualitative analysis of the hydrolysis products released from different oligosaccharides. For TLC analysis, 3 µL of reaction mixtures were loaded on silica gel 60 TLC plates (Merck, Germany) and separated with three consecutive runs in saturated TLC chambers using a solvent mixture of chloroform:acetate:water (6:7:1, *v*/*v*/*v*), fully drying the plates between each run. The analytes separated on the plate were visualized by spraying with 2.5 mL staining solution containing 1% aniline (*v*/*v*) and 1% (*w*/*v*) diphenylamine in acetone mixed with 0.1 volume 85% H_3_PO_4_ in a DESAGA ChromaJet DS20 TLC spray chamber (Sarstedt, Nümbrecht, Germany), followed by heating for 10 min at 120 °C.

For examining the influence of metal ions (CuCl_2_, NiSO_4_, FeCl_2_ 4H_2_O, ZnCl_2_, CoCl_2_, MnCl_2_ 4H_2_O, CaCl_2_ 2H_2_O, NaCl, KCl, MgCl_2_ 6H_2_O and MgSO_4_), denaturants (SDS and urea), chelator (EDTA) and arabinose, these substances (except arabinose) were incubated with enzyme (0.56 µM *Am*Araf51 or 0.16 µM *Am*Araf43) at final concentrations of 10 mM, 5 mM and 1 mM in 25 mM citrate phosphate buffer at room temperature for 2 h, including negative controls, which contained only individual additives or only an enzyme. The effect of arabinose on both enzymes was tested by incubating 67 mM, 133 mM, 266 mM or 533 mM arabinose with 0.56 µM *Am*Araf51 or 0.16 µM *Am*Araf43 for 2 h, respectively, and a negative control using water instead of arabinose was included. Upon incubation, 2 mM *p*NP-AF was added to the reaction mixtures and residual activities were measured with the colorimetric *p*NP assay described above. 

In order to determine synergistic effects and hydrolysis yields obtained during SBA degradation and WAX-RS degradation, the two α-l-arabinofuranosidases *Am*Araf51 and *Am*Araf43 were combined with two endoactive enzymes, *Pp*Abn43 (arabinanase from *Paenibacillus polymyxa* DSM 292) and M_Xyn10 (xylanase from metagenomic library screening), two enzymes available in our laboratory. At a substrate concentration of 5 g L^−1^ of SBA or WAX-RS, different combinations of the endo- and exoacting enzymes were incubated in 25 mM citrate phosphate buffer (pH 5.5) at 50 °C for 24 h. For stepwise enzyme digestions, the endocleaving enzyme (18 µg each, corresponding to 0.9 µM *Pp*Abn43 and 2.9 µM M_Xyn10) was first incubated with a substrate (SBA or WAX-RS, respectively) for 12 h, followed by heat inactivation by boiling for 10 min and thereafter adding the exo-acting enzyme (0.3 µM *Am*Araf51 or *Am*Araf43) and incubation for an additional 12 h. For simultaneous enzyme digestions, reaction mixtures with SBA were incubated for 24 h with either 0.3 µM *Am*Araf51 plus 0.9 µM *Pp*Abn43 or 0.3 µM *Am*Araf43 plus 0.9 µM *Pp*Abn43 and reaction mixtures containing WAX-RS were incubated for 24 h with either 0.3 µM *Am*Araf51 plus 2.9 µM M_Xyn10 or 0.3 µM *Am*Araf43 plus 2.9 µM M_Xyn10. Arabinose and xylose liberated at different time points (0 h, 1 h, 2 h, 6 h, 12 h, 13 h, 14 h, 18 h and 24 h) were quantified by HPAEC-PAD, referring to a standard curve obtained from a series of different arabinose/xylose concentrations (200 mg L^−1^, 100 mg L^−1^, 50 mg L^−1^, 25 mg L^−1^ and 12.5 mg L^−1^).

## 3. Results

### 3.1. Bioinformatic Analysis of AmAraf51 and AmAraf43

**Figure 1:**
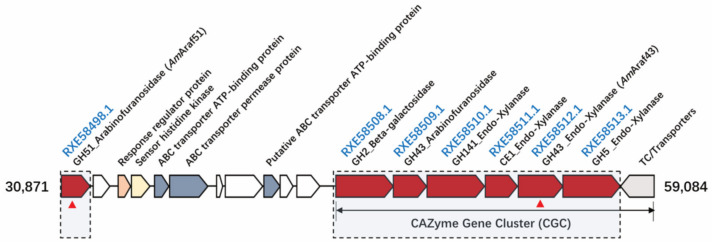
Gene organization of the putative CAZyme gene cluster on the genome of *A. mesophilus* N2K1 (functional annotation was performed by the dbcan database and Prokka Genome Annotation). The genes characterized in this study are marked with red triangles.

**Figure 2:**
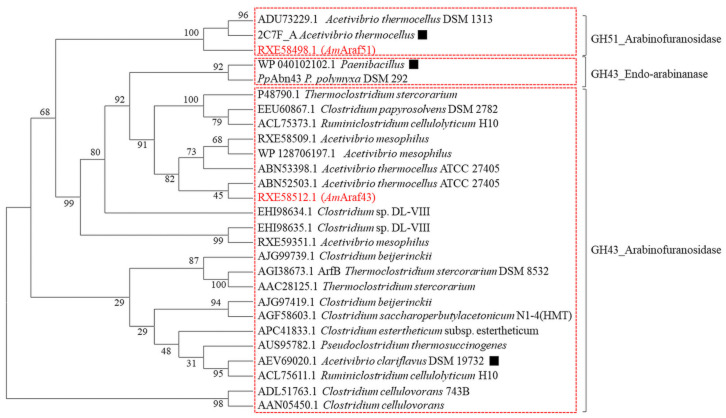
Phylogenetic tree of arabinosyl hydrolyases, including the exo-α-l-arabinofuranosidases (*Am*Araf51 and *Am*Araf43) and the endoarabinanase *Pp*Abn43 used in this study, and two other putative GH43 enzymes (encoded by RXE59509.1 and RXE59351.1) from *A. mesophilus* strain N2K1. An alignment of NCBI amino acid sequences was performed using the MUSCLE algorithm. A neighbor-joining (NJ) tree was then constructed in MEGAX and tested with 500 replicate bootstraps. Bootstrap values are listed near each branch. The functionally of the characterized arabinosyl hydrolyases are indicated with a black square symbol (■).

The genome of *Acetivibrio mesophilus* contains an approximately 26 kbp large cluster of colinearly arranged genes for various predicted glycoside hydrolases such as β-galactosidase (GenBank: RXE58508.1), arabinofuranosidases (GenBank: RXE58498.1 and RXE58509.1) and four endo-1,4-β-xylanases (consecutive genes with the GenBank number RXE58510.1, RXE58511.1, RXE58512.1 and RXE58513.1, respectively), which are related to hemicellulose and pectin substrate degradation (Figure 1). We focused on the GH51 and GH43 ORFs, which could encode putative arabinofuranosidases, i.e., RXE58498.1, RXE58509.1 and RXE58512.1. The RXE58509.1 ORF was not further studied, because it could not be expressed as a functional enzyme in *E. coli*. *Am*Araf51 is comprised of 509 amino acids with only one catalytic GH51 module (residues 297–501), no signal peptide was predicted. *Am*Araf51 showed 94.04% amino acid sequence identity with a putative GH51 arabinofuranosidase from *Acetivibrio thermocellus* (basonym *Clostridium thermocellum)* DSM 1313 (GenBank accession: ADU73229.1) and 93.84% identity with a characterized arabinofuranosidase from *A. thermocellus* (*Ct*Ara*f*51A, PDB accession: 2C7F_A) [28] (Figure 2), but the activities of *Ct*Ara*f*51A and *Am*Araf51 are significantly different. Another recently characterized GH51 arabinofuranosidase Clocl_2445 (GenBank accession: AEV69020), which is from an *Acetivibrio clariflavus* (syn. *Clostridium clariflavum*) strain, shared 28.66% identity with the GH51 enzyme from strain N2K1 (Figure 2). *Am*Araf43 on the other hand represents a 657 residue enzyme with an N-terminal signal peptide, a GH43 module (*Am*GH43A, residues 36–291), a module associated with some GH43 modules, GH43_C2 (pfam designation PF17851, beta xylosidase C-terminal concanavalin A-like domain) (*Am*GH43B, residues 321–504), a CBM6 module (residues 529–650) and a C terminal dockerin domain, which is separated from the rest of the enzymes by a short linker sequence. Two putative GH43 enzymes from *Clostridium* sp., with GenBank accession numbers ABN52503.1 and ABN53398.1 [29], have 52.45% and 52.67% identity with *Am*Araf43 (Figure 2).

### 3.2. Production and Purification of Recombinant AmAraf51 and AmAraf43

**Figure 3:**
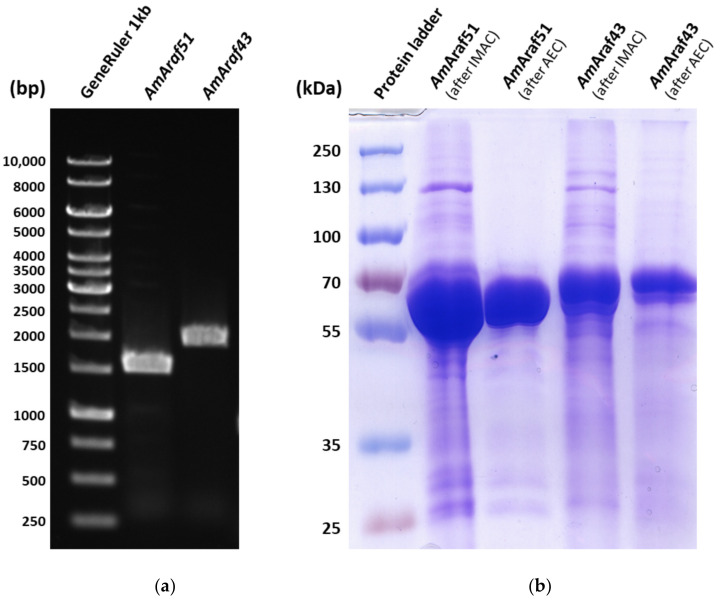
(**a**) PCR amplification of the genes *Am*Araf*51* and *Am*Araf*43* (without dockerin domain) Lane 1: GeneRuler 1kb, Lane2: *Am*Araf51 (1530 bps), Lane 3: *Am*Araf43 (1951 bps). (**b**) SDS-PAGE showing overexpression and purification of *Am*Araf51 and *Am*Araf43. Lane 1: Prestained protein standards; Lane 2: Sample (8.88 µg) of eluate with *Am*Araf51 (57.6 kDa) from immobilized metal affinity chromatography (IMAC) with a Ni-TED packed column; Lane 3: Sample (2.69 µg) with *Am*Araf51 after anion exchange chromatography (AEC); Lane 4: Sample (10.9 µg) of eluate with *Am*Araf43 (72.9 kDa) from IMAC with a Ni-TED packed column; Lane 5: Sample (2.74 µg) with *Am*Araf43 after anion exchange chromatography.

To investigate the properties of the putative α-l-arabinofuranosidase (*Am*Araf51, GenBank: RXE58498.1) and endo-1,4-β-xylanase (*Am*Araf43, GenBank: RXE58512.1) (initial enzyme function designations are based on automated Prokka Genome Annotation), the proteins were produced recombinantly in *Escherichia coli.* DNA fragments, containing the complete ORF encoding *Am*Araf51 and the ORF regions for modules *Am*GH43A, *Am*GH43B and CBM6 of *Am*Araf43, were amplified with PCR, in both cases with primer-based introduction of His_6_ tags, at the *N*-terminus of *Am*Araf51 and at the C-terminus of *Am*Araf43. The C-terminal dockerin domain of *Am*Araf43 was removed since such domains usually have no influence on the enzymatic activity and the initial experiments had indicated that its presence interferes with the affinity of *Am*Araf43 to bind to the nickel column during immobilized metal ion affinity chromatography (IMAC). The resulting PCR fragment of 1530 bps for *Am*Araf51 and 1951 bps for *Am*Araf43 (Figure 3a) were consistent with the theoretical values. The recombinant proteins were purified by IMAC with nickel columns followed by an anion exchange chromatography. The purity of the proteins was checked by SDS-PAGE, which also demonstrated that their migration was in agreement with the predicted molecular masses (57.58 kDa for *Am*Araf51, 72.92 kDa for *Am*Araf43) (Figure 3b). 

### 3.3. Enzyme Properties of AmAraf51 and AmAraf43 

**Figure 4:**
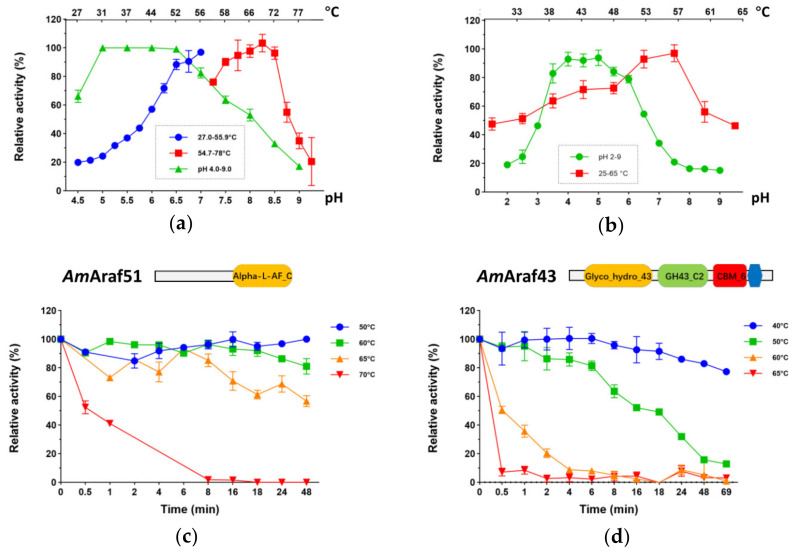
Temperature and pH dependence of *Am*Araf51 (**a**) and *Am*Araf43 (**b**), and thermoinactivation kinetics of *Am*Araf51 (**c**) and *Am*Araf43 (**d**). Relative activities were calculated from *p*NP-AF assay standard reactions (2 mM *p*NP-AF and 25 mM citrate phosphate buffer 20 min) with 6.2 nM *Am*Araf51 or 2.4 µM *Am*Araf43. The maximal activity was set as 100%. Error bars represent standard deviations (*n* = 3).

To optimize the reaction conditions, the temperature and pH profiles of *Am*Ara51 and *Am*Ara43 activity were determined using *p*NP-AF as a substrate. The highest activity under the given assay conditions (20 min assays, at pH 6.0 for both *Am*Araf51and *Am*Araf43) was recorded at temperatures of 70 °C for *Am*Araf51 and 57 °C for *Am*Araf43 (Figure 4a,b). *Am*Ara51 displayed over 60% activity in the range from 44 to 75 °C (Figure 4a), while *Am*Ara43 was more than 60% active at temperatures between 38 and 61 °C (Figure 4b). The optimal pH was between 5.0 and 6.5 for *Am*Araf51 and 4.0 and 5.0 for *Am*Araf43 (Figure 4a,b).

A critical factor regarding the applicability of enzymes in biotechnological processes is their resistance against thermoinactivation. Comparing the enzymes after a total of 48 h of incubation, the activity of *Am*Araf43 was reduced to 82.9% of the initial activity at 40 °C, while at 50 °C, the residual activity dropped to 15.8%. At temperatures above 60 °C, the residual activity after this time was less than 5% of the initial activity (Figure 4d). By contrast, *Am*Araf51 was more stable at higher temperatures. It maintained its activity throughout two days of incubation at 50 °C and retained about 80% or 60% of its initial activity after 2 days at 60 °C or 65 °C, respectively (Figure 4c).

### 3.4. Specific Activities on Various Polysaccharides and Kinetic Parameters

Various arabinose-containing substrates with different compositions and structures were used for cleavage specificity evaluation (Table 1). Both *Am*Ara51 and *Am*Ara43 showed activity against *p*NP-AF, a synthetic substrate for exo-α-l-arabinofuranosidase activity, which implies that both enzymes are exoacting α-l-arabinofuranosidases. With a specific activity of 1835.79 U mg^−1^, *Am*Araf51 showed the highest activity against this substrate (Table 1). *Am*Araf51 also displayed activity against arabinan, with 1.97 U mg^−1^ on debranched arabinan (DA) and 0.50 U mg^−1^ on sugar beet arabinan (SBA) (Table 1). The hydrolysis product released from either SBA or DA was only arabinose. Taken together, these results indicate that *Am*Araf51 is an exo-α-l-arabinofuranosidase. On the other hand, activity of *Am*Araf51 on soluble wheat arabinoxylan (WAX-RS) and insoluble wheat arabinoxylan (WAX-I) could hardly be detected, despite that both of these xylan substrates are decorated with arabinofuranosyl residues. In contrast, *Am*Araf43 activity on arabinan substrates was barely detectable, but the enzyme showed relatively high activity on arabinoxylan, especially WAX-RS (0.56 U mg^−1^) (Table 1). The product liberated during hydrolysis of these substrates by *Am*Araf43 was also only arabinose, no xylose or xylo-oligosaccharides were detected during 24 h of incubation with the WAX-RS substrates under the enzyme’s optimal reaction conditions (Figure S1b), further, *Am*Araf43 did not show any detectable activity against beechwood xylan as well (Table 1). *Am*Araf43 also cleaved *p*NP-AF, albeit with lower specific activity than *Am*Araf51. Therefore, *Am*Araf43 is also an exo-α-l-arabinofuranosidase, not a xylanase as indicated by the initial gene annotation. 

The kinetic constants of *Am*Araf51 for SBA and DA hydrolysis and of *Am*Ara43 for WAX-RS hydrolysis were calculated as the rate of arabinose equivalents released from the substrate polymers. Data fitted by nonlinear regression to the Michaelis–Menten model. *Am*Araf51 showed a lower K_m_ and V_max_ on SBA (K_m_ = 25.69 g L^−1^, V_max_ = 2.84 nmol min^−1^ µg^−1^) than on DA (K_m_ = 38.54 g L^−1^, V_max_ = 7.11 nmol min^−1^ µg^−1^) (Table 1, Figure S2a,b), suggesting *Am*Araf51 had slightly higher binding affinity for SBA than for DA. *Am*Araf43 revealed a similar K_m_ value (26.56 g L^−1^) for WAX-RS as *Am*Araf51 for SBA (K_m_ = 25.69 g L^−1^) (Table 1, Figure S2c).

### 3.5. Hydrolysis Patterns of AmAraf51 and AmAraf43 on Arabino-Oligosaccharides

**Figure 5:**
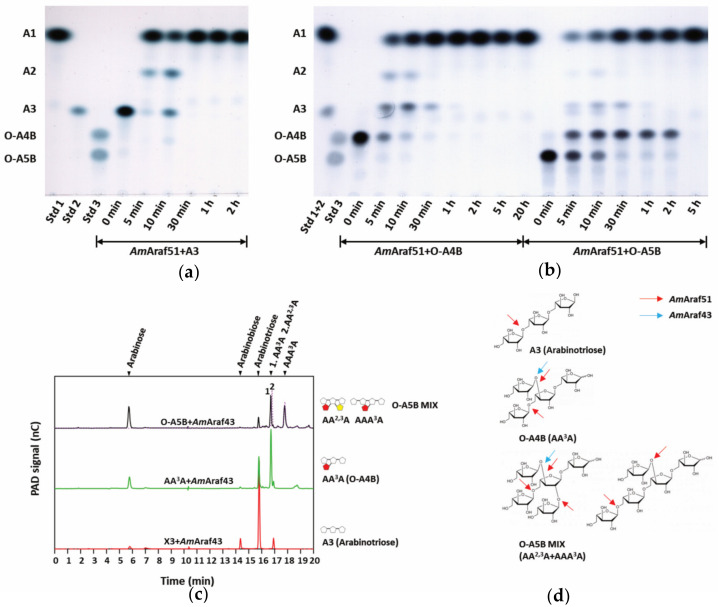
Time-course analysis of hydrolysis products of *Am*Araf51 with AOS by TLC (**a**) arabinotriose and (**b**) O-A4B or O-A5B as substrates. (**c**) HPLC analysis of hydrolysis products of *Am*Araf43 with AOS for 22 h of incubation (AA^3^A and AA^2,3^A are quite close, AA^3^A matches the left of the two peaks at a retention time of around 17 min). (**d**) Structure of three different AOS. Standard reaction was performed by incubating 85 nM *Am*Araf51 and 82 nM *Am*Araf43 with different AOXs at pH 6.0 and 60 °C for *Am*Araf51 and at pH 5.0 and 40 °C for *Am*Araf43 at different time periods. 2.5 µL of resulting hydrolysis products were separated three times on TLC plates using chloroform:acetate:water (6:7:1, *v/v/v*) as a solvent.

To gain more insight into the cleavage specificities of both enzymes, their activities on arabino-, arabinoxylo- and xylo-oligosaccharides (AOS, AXOS and XOS, respectively) with various branching patterns were analyzed by TLC or HPLC. The incubation of *Am*Araf51 with three different AOS led to their almost complete hydrolysis to arabinose within 1–5 h (Figure 5a,b): O-A4B represents a trisaccharide fragment of the α-1,5-linked arabinan backbone with a 1,3-linked arabinofuranosyl side group attached to the second arabinose; O-A5BMIX is comprised of two different AOS with arabinotriose and arabinotetraose as backbone structures, the former having two arabinofuranosyl substitutions at positions O-3 and O-2 of the second (counting from the non-reducing end) main chain arabinose, while the latter has an arabinofuranosyl branch at position O-3 of the third main chain arabinose (see Figure 5d). Therefore, the results with AOS as substrates indicated a broad cleavage specificity of *Am*Araf51 regarding the arabinofuranosyl linkage types. This enzyme was able to cleave 1,5-, 1,3- and 1,2-linkages. The time periods required for total hydrolysis of the three different AOS (A3 < O-A4B < O-A5BMIX) also indicated that the degree of polymerization (DP) and the complexity of the side chain substitution is negatively correlated with this enzyme’s cleavage efficiency, suggesting a preference of main chain (α-1,5-arabinosidic) bonds over glycosidic bonds linking arabinosyl side groups at O-3 and O-2 positions (Figure 5a,b). In contrast, incubation of *Am*Araf43 with α-1,5-linked arabinotriose did not result in the release of arabinose, whereas the minor amount of arabinose released from O-A4B and O-A5B indicates that *AmAraf*43 has weak activity towards 1,3- and 1,3- and/or 1,2-linkages, respectively, of these AOS but lacks 1,5-linkage-cleaving activity (Figure 5c).

### 3.6. Action Mode of the Enzymes on Linear and Branched Oligosaccharides

Both *Am*Araf51 and *Am*Araf43 were also tested for the degradation of linear XOS and arabinose-decorated XOS (AXOS) and the hydrolysis products were identified by HPAEC-PAD by comparing the retention times of the samples to that of the oligosaccharide standards. 

**Figure 6:**
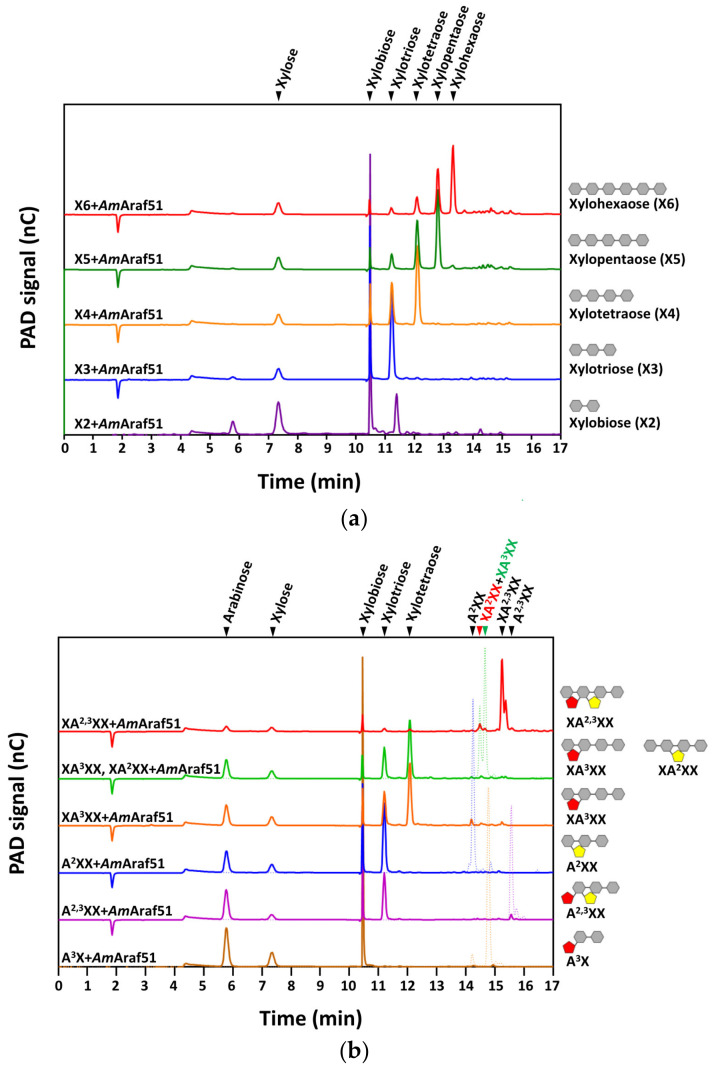

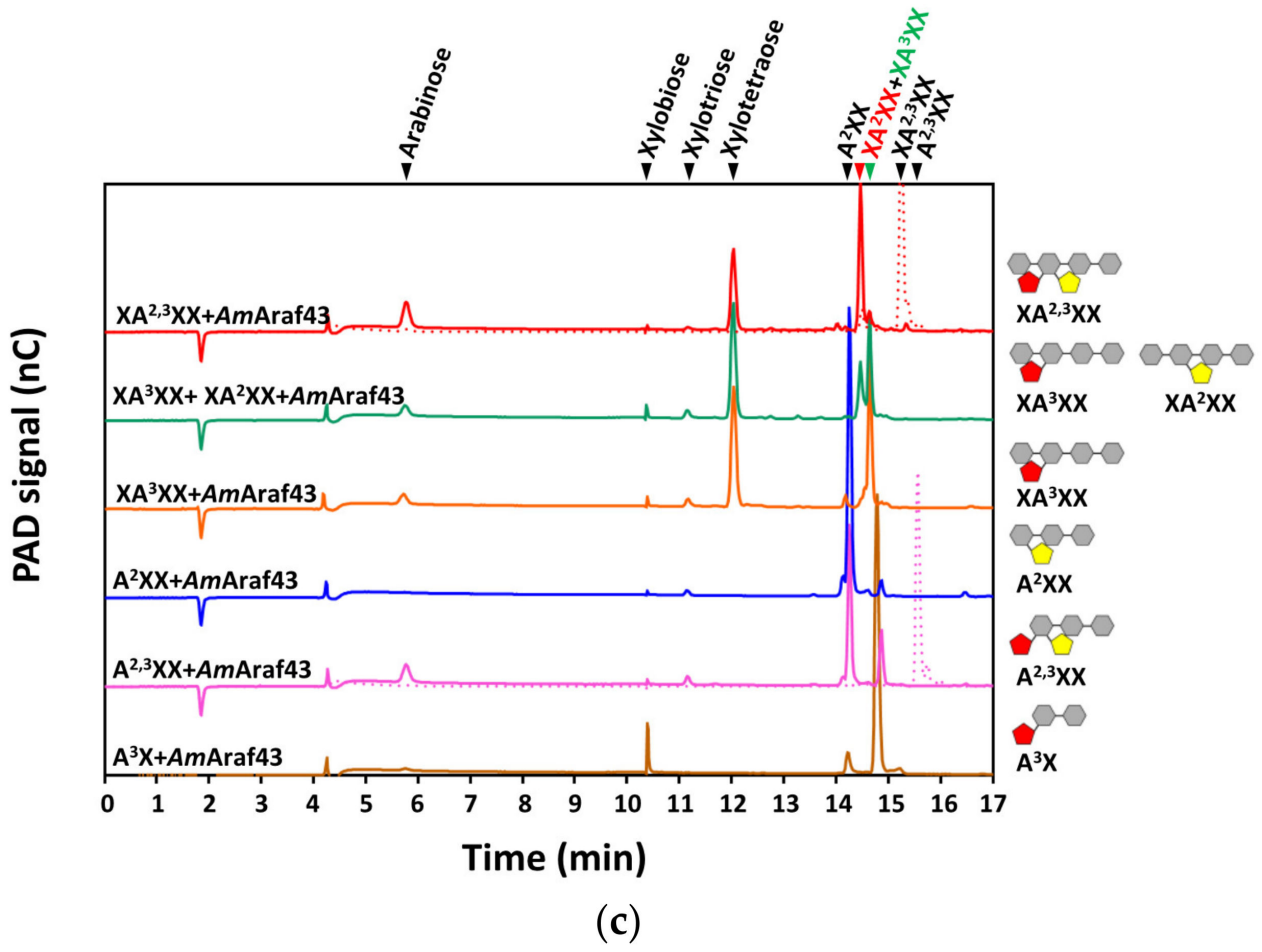
HPAEC-PAD analysis and structures of the hydrolysis products released from the (**a**) linear XOS by *Am*Araf51, (**b**) AXOS (branched XOS) by *Am*Araf51 and (**c**) AXOS by *Am*Araf43. Standard reactions were performed by incubating 85 nM *Am*Araf51 or 82 nM *Am*Araf43 with 0.25% of different XOS at 60 °C and pH 6.0 for 24 h for *Am*Araf51 and at 40 °C and pH 5.0 for 24 h for *Am*Araf43.

With linear XOS, the xylosidase side-activity of *Am*Araf51 could be confirmed, leading to cleavage of linear XOS from DP2 (degree of polymerization of 2) to DP6 and releasing XOS with lower DP along with a small amount of xylose (Figure 6a). When incubated with AXOS, *Am*Araf51 was able to liberate arabinose from 1,2- and 1,3-linked arabinosyl substitutions, and a β-xylosidase side-activity of *Am*Araf51 was evident from the liberation of a small amount of xylose after 24 h incubation of the enzyme with AXOS (Figure 6b). Putatively, the AXOS are first debranched by the arabinofuranosidase activity of *Am*Araf51, before a further, but far from complete, degradation of linear XOS takes place by the xylosidase activity (Figure 6b). In contrast, there was no xylosidase side-activity of *Am*Araf43, neither with the linear XOS nor with the branched AXOS.

*Am*Araf51 was able to cleave the arabinosylations off from singly modified AXOS, including XA^3^XX, XA^2^XX, A^2^XX and A^3^X, generating the corresponding linear XOS xylotretraose, xylotretraose, xylotriose and xylobiose, respectively, and its xylosidase side-activity cleaved off terminal xylose residues as just mentioned above (Figure 6b). *Am*Araf51 could also completely cleave off both arabinose moieties (1,2- and 1,3-linked) from the terminally twofold-arabinosylated A^2,3^XX without the appearance of monosubstituted A^3^XX or A^2^XX after 24 h. However, XA^2,3^XX, which contains an internal twofold arabinose-substituted xylose, only a minor amount of arabinose and xylose can be detected in the hydrolysis product (Figure 6b).

In contrast, *Am*Araf43 could cleave off the 1,3-linked arabinofuranosyl residue from either terminally or internally diarabinosylated A^2,3^XX and XA^2,3^XX, thus generating the hydrolysis products A^2^XX and XA^2^XX, respectively, but the 1,2-linked arabinose side chain in these products could not be removed completely (Figure 6c). Besides, *Am*Araf43 also showed incomplete removal of the single 1,3-linked arabinosyl residue from terminally or internally decorated A^3^X and XA^3^XX, thus only part of these oligosaccharides was converted to X2 and X4, respectively, in the corresponding reactions (Figure 6c).

### 3.7. Influence of Metal Ions on the Activity of Enzymes

The influence of various metal ions was checked under the standard *p*NP-AF assay conditions. For *Am*Araf51, slight activity increased between about 30% and 60% were observed with 10 mM Ca^2+^, 5 mM/10 mM Na^+^, 1 mM Mn^2+^ and 5 mM K^+^, and with 5–10 mM urea or EDTA (Figure S3a). Cu^2+^, Ni^2+^ and Fe^2+^ at all tested concentrations, Zn^2+^ at 5 mM or more and Co^2+^ or Mn^2+^ at 10 mM strongly inhibited the activity of *Am*Araf51 (Figure S3a). For *Am*Araf43, especially Mn^2+^ at 5 and 10 mM had a stimulating effect of about 40–60% (Figure S3b). Besides, both of *Am*Araf51 and *Am*Araf43 exhibited an arabinose tolerance of approximately 530 mM (Figure S3c).

### 3.8. Synergistic Action between AmAraf51, AmAraf43 and PpAbn43, M_Xyn10

**Figure 7:**
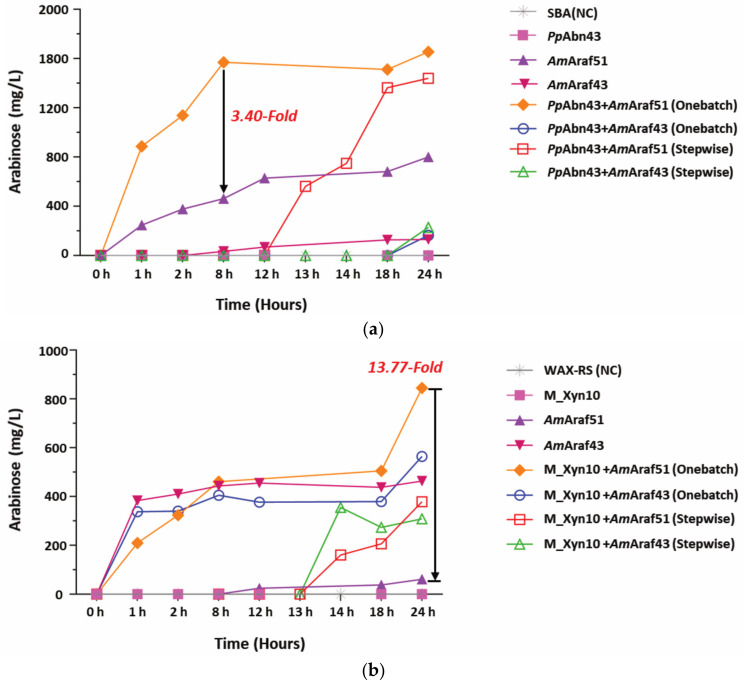

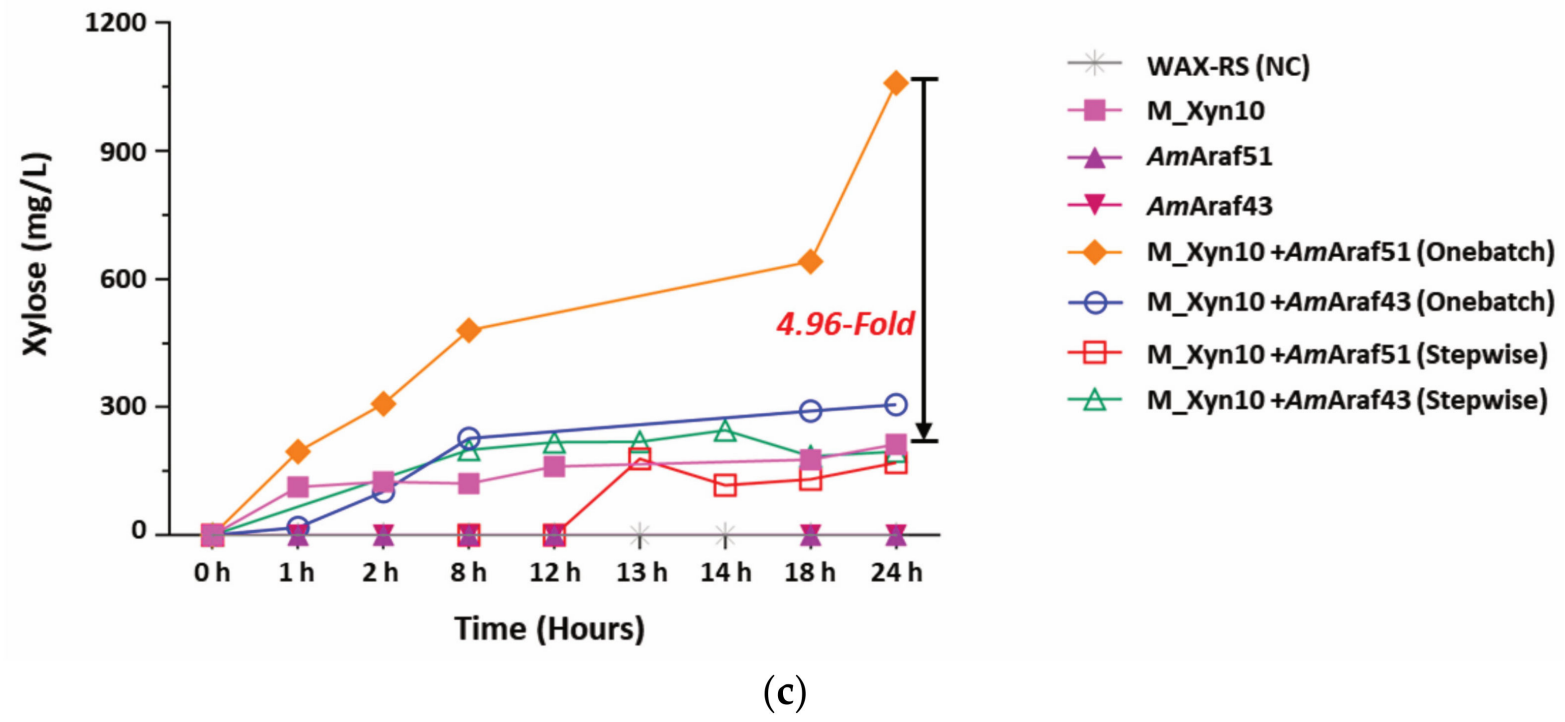
Hydrolysis of 0.5% (*w*/*v*) sugar beet arabinan (SBA) or wheat flour arabinoxylan (WAX-RS) by different combinations of endoactive enzymes (0.90 µM *Pp*Abn43 or 2.9 µM M_Xyn10) and exoactive enzymes (0.3 µM *Am*Araf51 or *Am*Araf43) at 50 °C and pH 5.5 over time. (**a**) Arabinose released from SBA. (**b**) Arabinose released from WAX-RS. (**c**) Xylose released from WAX-RS. The concentrations of arabinose and xylose in the reactions were quantified by HPAEC-PAD analysis.

*Am*Araf51 could not efficiently cleave the arabinofuranosyl side chains from WAX, whereas it was able to efficiently hydrolyze the arabinose homopolymers SBA and DA. In addition, interestingly, besides its major activity as an exoarabinofuranosidase, *Am*Araf51 revealed a β-xylosidase side-activity. *Am*Araf43 on the other hand had almost no activity on arabinan substrates but displayed relatively high activity on arabinoxylan, especially WAX-RS. Based on their substrate specificities, we hypothesized that these two arabinofuranosidases, *Am*Araf51 and *Am*Araf43, could be combined with endocleaving polysaccharide hydrolases, i.e., endoarabinanase and endoxylanase, for the synergistic degradation of arabinan and xylan, respectively, such as SBA and WAX and thus could be of use as components of defined enzyme cocktails for polysaccharide monomerization. This was tested with the endoarabinanase *Pp*Abn43 from *Paenibacillus polymyxa* and the endoxylanase M_Xyn10 from a metagenomic library screening. *Pp*Abn43 hydrolyzed SBA to produce mainly AOS but did not release arabinose (Figure 7a, Figure S1a), while M_Xyn10 degraded WAX-RS into XOS and AXOS and simultaneously released some xylose (Figure 7c, Figure S1b). When a cocktail of *Pp*Abn43 and *Am*Araf51 was added to SBA, the production of arabinose increased markedly compared with each enzyme working alone, leading to an about 2.1-fold higher arabinose concentration after 24 h of SBA treatment with the enzyme mixture compared to SBA incubation with *Am*Araf51 alone (Figure 7a). Stepwise treatment, first with *Pp*Abn43 (which released no detectable monomeric arabinose), followed by *Am*Araf51 addition after 12 h yielded slightly less arabinose than at the simultaneous incubation with both enzymes for 24 h (Figure 7a). Since *Am*Araf43 did not show strong activity against arabinan or AOS (Table 1, Figure 5c), only very little arabinose was released from SBA with its aid in combination with *Pp*Abn43, neither during simultaneous incubation with *Pp*Abn43 and *Am*Araf43 nor via *Am*Araf43 addition after pretreatment with *Pp*Abn43 (Figure 7a).

Possible synergistic effects of the arabinofuranosidases *Am*Araf51 and *Am*Araf43 were also studied in combination with endoxylanase M_Xyn10 using wheat arabinoxylan (WAX-RS) as the substrate. In this case, we quantified the yield of both arabinose and xylose. A 24 h incubation of WAX-RS simultaneously with M_Xyn10 and *Am*Araf51 resulted in a 13.77-fold higher yield of arabinose (845.1 mg L^−1^) compared with *Am*Araf51 alone (61.4 mg L^−1^) (Figure 7b). The stepwise treatment with the same combination of enzymes, i.e., incubation of WAX-RS first with M_Xyn10 followed by *Am*Araf51, generated 378.6 mg L^−1^ arabinose (Figure 7b). *Am*Araf43, which was shown to have higher activity than *Am*Araf51 on WAX-RS (Table 1), released 463.6 mg L^−1^ arabinose after 24 h without the addition of the endoxylanase M_Xyn10. There was slightly more arabinose measured after simultaneous incubation of *Am*Araf43 together with M_Xyn10 (564.5 mg L^−1^) (Figure 7b). The yield of arabinose after a two-step treatment of WAX-RS with M_Xyn10 followed by *Am*Araf43 was substantially lower (308.5.0 mg L^−1^) (Figure 7b). With respect to xylose production, the combined and simultaneous action of M_Xyn10 and *Am*Araf51 resulted in 1059.0 mg L^−1^ xylose, while the single-enzyme treatment with M_Xyn10 alone released about 5 times less xylose (213.5 mg L^−1^) (Figure 7c).

## 4. Discussion

In nature, bacteria degrade recalcitrant polymeric carbohydrates by carbohydrate-active enyzmes [11], which include glycoside hydrolases, polysaccharide lyases, carbohydrate esterases and glycosyl transferases plus a range of auxiliary enzymes [30]. Often, CAZyme-encoding genes are organized in clusters, frequently containing genes for sugar transport and regulation, in particular in bacteria specialized on the degradation of complex plant cell wall polysaccharides [31]. Some saccharolytic bacteria from the group of clostridia have evolved special multifunctional protein complexes named cellulosomes, which integrate numerous cellulases, hemicellulases and accessory enzymes assembled on non-catalytic scaffold proteins and form highly efficient (hemi)cellulolytic systems [32]. For example, the genome of *Ruminiclostridium cellulolyticum* contains a 32-kb *xyl-doc* gene cluster encoding 14 putative cellulosome enzymes, which are predicted to be involved in hemicellulose degradation [21], four of them were characterized to have α-l-arabinofuranosidase activity. In the genome of the recently isolated *Acetivibrio mesophilus* (basonym *Hungateiclostridium mesophilum*) strain N2K1 [22], the gene cluster we focused on here, contains two genes annotated to encode for α-l-arabinofuranosidases (RXE58498.1 and RXE58509.1, but the latter without corresponding enzymatic activity) and other genes predicted to be involved in hemicellulose degradation including genes for a putative β-galactosidase (RXE58508.1) and four putative endoxylanases arrayed downstream of gene RXE58509.1 (including RXE58510.1, RXE58511.1, RXE58512.1 and RXE58513.1), all in the same orientation (Figure 1). Upstream of these genes, a gene for a putative GH51 α-l-arabinofuranosidase (*Am*Araf51) without a signal peptide was found (RXE58498.1) separated from the other CAZyme genes by several genes encoding transport and regulatory proteins.

Due to the predicted functions, it can be suggested that the CAZymes gene cluster is involved in hemicellulose breakdown and uptake of the breakdown products, which is in agreement with the ability of strain N2K1 to decompose complex lignocellulosic substrates such as cellulose, wheat arabinoxylan, oat spelt xylan and sugar beet pulp [22]. All the genes between RXE58508.1 and RXE58513.1 contain C-terminal dockerin domain- and *N*-terminal signal peptide-encoding sequences (signal peptides predicted with SignalP-5.0), indicating that these genes encode extracellular cellulosomal proteins. Since strain N2K1 is a poor utilizer of externally added monosaccharides [22], the co-occurrence of transport protein-encoding genes in the hemicellulose degradation gene cluster suggests that oligosaccharides are transported into the cell before being further hydrolyzed to monomeric sugars. Based on the lack of a signal peptide, *Am*Araf51 (product of RXE58498.1) can be assumed to be an intracellular protein and may be involved in the hydrolysis of arabinose-containing oligosaccharides upon their internalization into the cytoplasm, like other cytosolic arabinofuranosidases [33]. However, a (partially) extracellular role could still be possible, since for example the extracellular proteome analysis of a *B. subtilis* strain revealed a significant number of proteins without a signal peptide in the culture supernatant [34], which may have been released by alternative secretion mechanisms or by bacterial cell lysis [35].

Arabinofuranosidases play an important role in the saccharification of arabinose-containing substrates, including arabinan and arabinoxylan. WAX-RS and WAX-I have almost the same Ara*f*/Xyl*p* ratio of 38:62 and 36:51, respectively, but WAX-I degradation is more difficult due to the presence of ferulic acid cross-links and the acetylation of xylose residues, whereas WAX-RS is extracted under alkaline conditions resulting in the removal of the ferulic acid residues [21,25]. Sugar beet arabinan as used in our study contains arabinofuranose residues attached to around 60% of O-3 positions of the α-1,5-linked arabinan backbone units and less frequently with α-1,2 arabinofuranose [36], while debranched arabinan exhibits a backbone with a very low degree of substitution. *Am*Araf51 and *Am*Araf43 have totally opposite preferences toward arabinan or arabinoxylan substrates: *Am*Araf51 has a much higher specific activity against arabinan substrates, especially debanched arabinan (decreasing order of activities on linear arabinan (LA) > debranched arabinan (DA) > sugar beet arabinan (SBA)), demonstrating that *Am*Araf51 has a strong ability to cleave α-1,5-arabinofuranosyl linkages of the arabinan backbone. On the other hand, its activity, measured against the arabinoxylans WAX-I and WAX-RS, was at the detection limit of the assay used. The substrate (arabinan) backbone preference of *Am*Araf51 also seems to be reflected by its hydrolysis reactions with oligosaccharides (also see below). *Am*Araf51 efficiently cleaved off the di-arabinosyl substitutions on interior residues of an arabinose-based backbone (AA^2,3^A), but not of a xylopyranosyl backbone (XA^2,3^XX) (Figure 5b and Figure 6b). Since *Am*Araf51 furthermore cleaves off the arabinosyl branches of AOS, this enzyme is proposed to play a role in the utilization of arabinan and AOS by strain N2K1.

*Am*Araf43 exhibited a converse order of substrate preferences compared with *Am*Araf51, with the highest specific activity towards WAX-RS, which was followed by WAX-I, while almost no activity was detected with pectic arabinan. *Am*Araf43 lacks the ability to cleave the arabinan backbone. This enzyme, which was originally annotated to be a putative endoxylanase, also did not cleave the xylan backbone, yielding only arabinose but no xylo-oligosaccharides as hydrolysis products after incubation with WAX. Thus, *Am*Araf43 can be classified as an exo-α-l-arabinofuranosidase, not surprisingly demonstrating that automated annotation can be misleading with regard to GH43 enzymes. Analogously, the enzyme encoded by gene *axb8* from *A. thermocellus* (*C. thermocellum*) strain B8 (GH43_29) was predicted to be an exoarabinofuranosidase but only released xylose instead of arabinose from WAX [37]. In general, the specificity and activity of arabinofuranosidases on arabinoxylans are likely mostly related to molecular details of their substrate binding sites and the presence of carbohydrate-binding modules such as CBM6, CBM35, CBM13 or CBM42, which can enable the enzymes’ attachment to the xylose polymer [38,39]. The CBM6 module of *Am*Araf43, thus, may influence the binding ability of the enzyme to xylans, but this has not yet been experimentally demonstrated. Like *Am*Araf43, *Ct*Abf43A from *C. thermocellum* has been reported to only liberate arabinose from side chains of arabinoxylan, but not from sugar beet arabinan [40]. Structural analysis of *Ct*Abf43A showed that this enzyme contained a long substrate-binding cleft that is complementary to the xylan backbone but not to an arabinan backbone [29]. *Am*Araf43 from N2K1 shared 59.20% amino acid identity with *Ct*Abf43A, therefore its molecular determinants of substrate specificity may also apply to *Am*Araf43.

We further used defined arabinosylated arabino- and xylo-oligosaccharides to evaluate the action mode of the arabinofuranosidases of this study. Both enzymes showed cleavage activity against arabinose residues at the O-2 and/or O-3 position, but the precise nature of the side chain substitution affected the catalytic efficiency significantly. With AOS, the time required for *Am*Araf51 to completely hydrolyze the substrate was negatively correlated with the complexity of the substrate oligomer (A3 > AA^3^A > AA^2,3^A + AA^3^AA), while *Am*Araf43 revealed the opposite preference (AA^2,3^A +AA^3^AA > AA^3^A > A3), an activity towards α-1,5-arabinosyl linkages, as in A3 as a substrate, was barely detectable. Both enzymes are able to remove arabinose moieties from linear and branched XOS, but the activity of *Am*Araf51 on internally twofold arabinosylated XA^2,3^XX is very weak, in contrast to its high activity towards internally double substituted arabinotriose (AA^2,3^A) again demonstrating this enzyme’s preference for the arabinose-based backbone. Based on this observation, the weak activity of *Am*Araf51 against arabinose-substituted XOS can be classified into the arabinofuranosidase type AXHs-m,d, which has a dual activity on terminally and/or internally located monosubstituted and double substituted xylose [21]. In comparison, *Am*Araf43 has a strong preference to remove the arabinose at O-3 position in double substituted xylose residues, no matter whether this is a terminal or an internal xylose (XA^2,3^XX, A^2,3^XX), while single 1,3-side chain substitutions are less preferred, which matches the characteristics of AXHs-m,d3 type arabinofuranosidases [21]. Further, with linear XOS as a substrate, *Am*Araf51 also showed β-xylosidase side-activity. This is also the case with side chain modified XOS, where we found various linear XOS in the hydrolysis products. This kind of bifunctional cleavage of arabinosidic and xylosidic linkages was also found for other enzymes such as a GH51 representative from *Alicyclobacillus* sp. A4, which displayed a combination of arabinofuranosidase and endoxylanase activities releasing arabinose, xylobiose, xylotriose and xylotetraose from water-soluble wheat arabinoxylan [41]. Additionally, another GH51 arabinofuranosidase from *Paenibacillus* sp. THS1 can hydrolyze not only side-chain but also main-chain glycosidic bonds in heteroxylans [42]. It has been described that the GH51 Abf from *G. stearothermophilus* T-6 can bind xylopyranosidic substrates in its active site [43], although in this case xylanase activity was not determined. The physiological relevance of the cleavage of β-xylosidic bonds in xylans or XOS by GH51 arabinofuranosidases remains to be elucidated. In the case of *Am*Araf51 of the present study, which due to the lack of a predicted N-terminal signal pepetide is postulated to be an intracellular enzyme, it is possible that both the arabinofuranosidase and the β-xylosidase activities could be of relevance during the decomposition of internalized AXOS/XOS and AOS originating from xylan and arabinan, respectively.

Enzyme thermostability is a balancing act between enzymatic activity and stability, which determines the overall efficiency over time and therefore is one of the most crucial factors that determines the temperature range of usage in industrial applications. *Am*Araf51 displayed superior performance compared to *Am*Araf43 in terms of overall resistance against thermoinactivation and stability at the temperature of its highest activity. The catalytic module of GH51 has a N-terminal (β/α)_8_-barrel architecture followed by a β-sandwich domain with an unknown function [1]. Enzymes with a (β/α)_8_-barrel architecture from thermophiles can be stabilized by various means, such as an increased association state, additional salt bridges or hydrogen bonds [44], leading to half-lives of GH51 enzymes ranging from hours up to several days at high temperature, for example, Abf51 from *A. clariflavus* DSM 19732 had a half-life of seven days at its optimal catalyzing temperature of 60 °C [45]. In contrast, GH43 enzymes, with a fivefold β–propeller architecture, are less studied in detail with regard to their thermostability compared with GH51 [14]. 

As both *Am*Araf51 and *Am*Araf43 display activity with polysaccharide substrates, we finally discuss the potential of these enzymes as components of enzyme cocktails for the decomposition of plant biomass in enzyme reactors. The total hydrolysis of heteroxylan and pectic arabinan requires exoacting accessory enzymes, such as arabinofuranosidase, to generate debranched intermediates. Thereby, they are better accessible for endoacting enzymes such as endoxylanase (for arabinoxylan) and endoarabinanase (for SBA). In recent years, various studies have focused on the use of enzyme cocktails for biomass degradation. For example, Geng et al., reported that the presence of Abf51 from *Acetivibrio clariflavus* (*Hungateiclostridium clariflavum*) DSM 19732 [45] dramatically improved the saccharification level of arabinoxylan (18.5 g L^−1^) up to six times along with a GH11 β-1,4-xylanase (XynA) from *Thermomyces lanuginosus* [46] and a GH43 β-1,4-xylosidase (Xyl43C) from *Clostridium clariflavum* [47]. On the other hand, Bouraoui et al. showed that the combination of a GH51 arabinofuranosidase and a GH11 endoxylanase did not result in synergy [42]. A GH11 xylanase may not be the best combination with a GH51 arabinofuranosidase because GH11 xylanases produce cleavage products with double substitutions mostly on internal rather than terminal xylopyranosyl residues [48] (Figure S1c), such decorations largely resist hydrolysis by GH51 and other arabinofuranosidases [18], as was also observed by us, i.e., the catalytic efficiency of *Am*Araf51 was very low with XA^2,3^XX. Recently, a GH51 arabinofuranosidase (*Xac*Abf51) was reported to be able to cleave arabinofuranosyl linkages of internal disubstitutions of xyloparanosyl units, which can be attributed to the presence of a pocket arranged near to subsite-1 that can accommodate a second arabinofuranosyl group [15]. In general, the hydrolytic ability of GH51 arabinofuranosidases (also including *Am*Araf51) against double substitutions on terminal xylopyranosyl residues such as in A^2+3^XX, suggests a superior activity on GH10- rather than GH11-xylanase-derived arabinoxylan degradation products, of which some have arabinosyl disubstitutions on terminal xylopyranosyl residues [48]. In agreement, our WAX-RS degradation experiments (Figure 7) showed the synergistic effects of *Am*Araf51 and M_Xyn10, which in combination increased the yields of arabinose almost 14-fold and of xylose almost 5-fold compared with single enzyme treatments with only *Am*Araf51 or only M_Xyn10, respectively. Furthermore, the one batch treatment of SBA with *Am*Araf51 and *PpA*bn43 enhanced the liberation of arabinose by 3.4 fold after 6 h compared with *Am*Araf51 alone, showing that *Am*Araf51 may also be useful in sugar beet arabinan monomerization when combined with an endoarabinanase. Finally, a further factor thats may affect the yields of enzymatic substrate conversion is inhibition of the enzymes by cleavage products. To this end, both *Am*Araf51 and *Am*Araf43 compare favorably with other arabinofuranosidases (IC_50_ = 100–500 mM) [49,50], as their *p*NP-α-l-arabinofuranoside-cleaving activity was not significantly reduced by up to 533 mM of the product arabinose (Figure S3c).

## 5. Conclusions

*Acetivibrio mesophilus* is a cellulolytic organism with hemicellulose degradation ability towards arabinose-containing substrates such as different xylans and sugar beet pulp. This work elucidated the enzymatic characteristics and differences of two arabinofuranosidases *Am*Araf43 and *Am*Araf51, from *A. mesophilus*, highlighting their specific activities towards AX and SBA, respectively. *Am*Araf51, a thermostable enzyme, with its broad cleavage ability towards single and double substitutions in AOS and its β-xylosidase side activity as seen from its activity on AXOS/XOS, suggests it may be involved in the decomposition of various arabinan- and xylan-derived oligosaccharides. In comparison, *Am*Araf43 preferentially removes arabinosyl residues from the O-3 position in disubstituted xylose residues. Both *Am*Araf51 and *Am*Araf43 in addition displayed activity with polysaccharide substrates, i.e., arabinan and arabinoxylan, respectively, and in particular *Am*Araf51 revealed significant synergistic effects regarding arabinose liberation from arabinan and arabinoxylan when administered together with an endoarabinanase or an endoxylanase, respectively. Our further work will focus on the study of enzyme cocktail synergism during degradation of recalcitrant plant biomass, especially SBP. In addition to investigating the enzymatic breakdown of purified poly- and oligosaccharides, it will be important to study in more depth the potential role of accessory enzymes, such as arabinofuranosidases, in the efficient and complete decomposition of plant biomass. This represents a complex interwoven matrix of different polymers. It will be a large challenge to elucidate the multiple synergistic interactions between several different simultaneously acting enzymes in order to achieve the final goal of generating low-cost defined enzyme cocktails for highly efficient monomerization of complex agricultural residues such as SBP.

## Figures and Tables

**Table 1:** Specific activities and kinetic parameters of *Am*Araf51 and *Am*Araf43 on different substrates.

Specific Activity: U/mg								
	LA	DA	SBA	WAX-I	WAX-RS	BWX	SBP	*p*NP-AF
*Am*Araf51	4.3 ± 0.24	1.97 ± 0.15 K_m_ = 38.54 V_max_ = 7.11	0.50 ± 0.05 K_m_ = 25.69 V_max_ = 2.84	NA	NA	-	NA	1835.79 ± 15.02
*Am*Araf43	-	-	NA	0.03 ± 0.01	0.56 ± 0.04 K_m_ = 26.56 V_max_ = 1.64	-	-	0.41 ± 0.03

Enzyme activity on 0.5% of different substrates was determined by DNS reducing sugar assay. One unit of activity was defined as the amount of enzyme to release 1 µmol of l-arabinose equivalent per minute under the optimal temperature and pH. LA: Linear arabinan; DA: debranched arabinan; SBA: sugar beet arabinan; WAX-I: wheat arabinoxylan (insoluble); WAX-RS: wheat arabinoxylan for reducing sugar assay; BWX: beechwood xylan; SBP: sugar beet pulp; *p*NP-AF: *p*NP-arabinofuranoside; NA: not available. “-’’: no activity detected; U/mg = µmol mg^−1^ min^−1^; K_m_ = g L^−1^; V_max_ = nmol min^−1^ µg^−1^.

## Data Availability

All data are available from the corresponding author upon reasonable request.

## Supplementary Materials

The following are available online at https://www.mdpi.com/article/10.3390/microorganisms9071467/s1, Figure S1: HPLC analysis of the hydrolysis of 0.5% (*w/v*) (a) sugar beet arabinan or (b) wheat flour arabinoxylan (for reducing sugar assay) by different combination of endoactive enzymes (0.90 µm *Pp*Abn43 or 2.9 µm M_Xyn10) and exoactive enzymes (0.3 µm *Am*Araf51 or 0.3 µm *Am*Araf43) at 50 °C and pH 5.5 for 24 h incubation. (c) Scheme of arabinoxylan degradation by the synergistic effect of M_Xyn10 and *Am*Araf51. Figure S2: Determination of kinetic parameters of (a) *Am*Araf51 degrading debranched arabinan, (b) sugar beet arabinan and (c) *Am*Araf43 degrading wheat flour arabinoxylan (for reducing sugar assay). Standard reactions (25 mM citrate phosphate buffer, at pH 6.0 and 60 °C for *Am*Araf51 and pH 5.0 and 50 °C for *Am*Araf43, 2 h incubation) with three different enzyme concentrations and different substrate concentrations were applied. K_m_ and V_max_ were determined by Microsoft Excel Solver. Error bars represent the standard deviation of duplicates. Figure S3: Influence of metal ions, protein denaturants and metal chelator on the activity of (a) *Am*Araf51 and (b) *Am*Araf43. (c) Effects of arabinose on *Am*Araf51 and *Am*Araf43. Standard reaction (2 mM *p*NP-AF, 25 mM citrate phosphate buffer pH 6.0 and 4.5 for *Am*Araf51 and *Am*Araf43, respectively, 20 min) were performed as described in the materials and methods, using 0.56 µM *Am*Araf51 and 0.16 µM *Am*Araf43 with 1 mM, 5 mM and 10 mM salt solution, respectively, or with 67 mM, 133 mM, 266 mM and 533 mM arabinose, respectively. Activities are expressed relative to reactions without additional ions/arabinose (H_2_O). Error bars show the standard deviation of triplicates. Table S1: Arabino-oligosaccharides (AOS) and arabinoxylo-oligosaccharides (AXOS) used in this study. The oligosaccharides were named following the Megazyme Nomenclature.
