# Supplementary material for: Characterization of Two α-l-Arabinofuranosidases from Acetivibrio mesophilus and Their Synergistic Effect in Degradation of Arabinose-Containing Substrates
Source: Microorganisms. 2021 Jul 8;9(7):1467. doi: 10.3390/microorganisms9071467 (PMC8307384; doi:10.3390/microorganisms9071467)
Supplement: Supplementary file 1 [file microorganisms-09-01467-s001.zip › microorganisms-1258343-supplementary.pdf]

Supplementary Table S1. Arabino-oligosaccharides (AOS) and arabinoxylo-oligosaccharides (AXOS) used in this study. The oligosaccharides were named following the Megazyme Nomenclature.

Product code	Name	Structure
O-A3X	3^3-α-L-Arabinofuranosyl-xylobiose (A^3X)	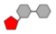
O-A2XX	2^3-α-L-Arabinofuranosyl-xylotriose (A^2XX)	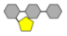
O-XA3XX	3^3-α-L-Arabinofuranosyl-xylotetraose (XA^3XX)	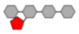
O-A2X3	2^2,3^2-di-α-L-Arabinofuranosyl-xylotriose ($A^{23}XX$)	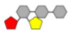
O-XA23XX	2^2,3^2-di-α-L-Arabinofuranosyl-xylotretraose ($XA^{23}XX$)	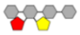
O-XAXX MIX	3^3-α-L- plus 2^3-α-L-Arabinofuranosyl-xylotetraose (XA^3XX/XA^2XX) mixture	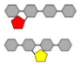
O-A4B	3^2-α-L-Arabinofuranosyl-(1,5)-α-L-arabinotriose (AA^3A)	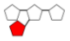
O-A5BMIX	2^2,3^2-di-α-L-Arabinofuranosyl-(1,5)-α-L-arabinotriose plus 3^2-α-L-arabinofuranosyl-(1,5)-α-L-arabinotetraose ($AA^{23}A$+AAA^3A)	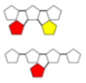

*Grey hexagon represents xylopyranosyl residue, white pentagon represents arabinofuranosyl residue, red pentagon means 1,3- linked arabinofuranosyl residue, yellow pentagon means 1,2-linked arabinofuranosyl residue.

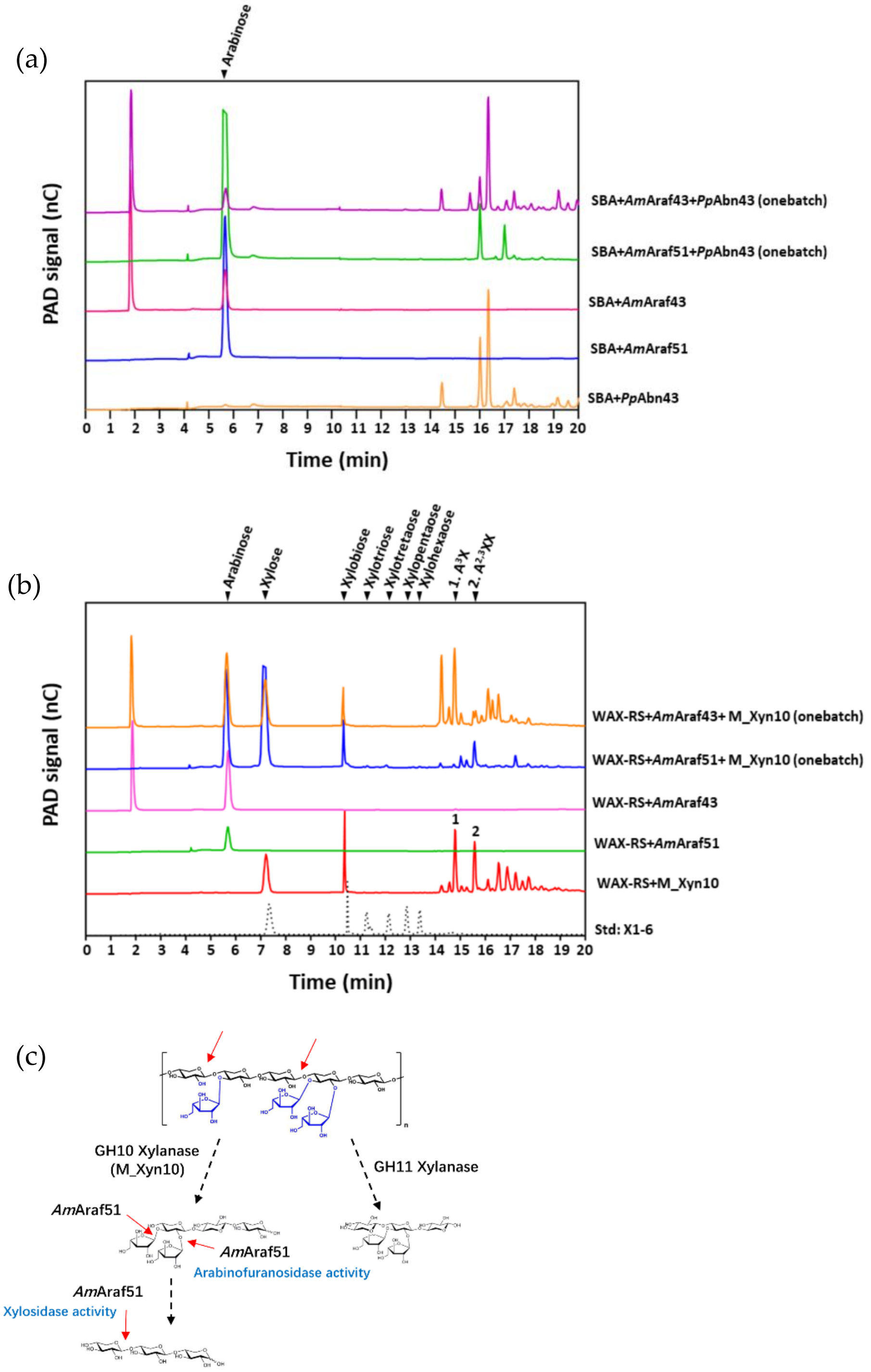

Supplementary Figure S1. HPLC analysis of the hydrolysis of 0.5% (w/v) (a) sugar beet arabinan or (b) wheat flour arabinoxylan (for reducing sugar assay) by different combination of endo-active enzymes (0.90 μm *Pp*Abn43 or 2.9 μm M_Xyn10) and exo-active enzymes (0.3 μm *Am*Araf51 or 0.3 μm *Am*Araf43) at 50 °C and pH 5.5 for 24 h incubation. (c) Scheme of arabinoxylan degradation by the synergistic effect of M_Xyn10 and *Am*Araf51.

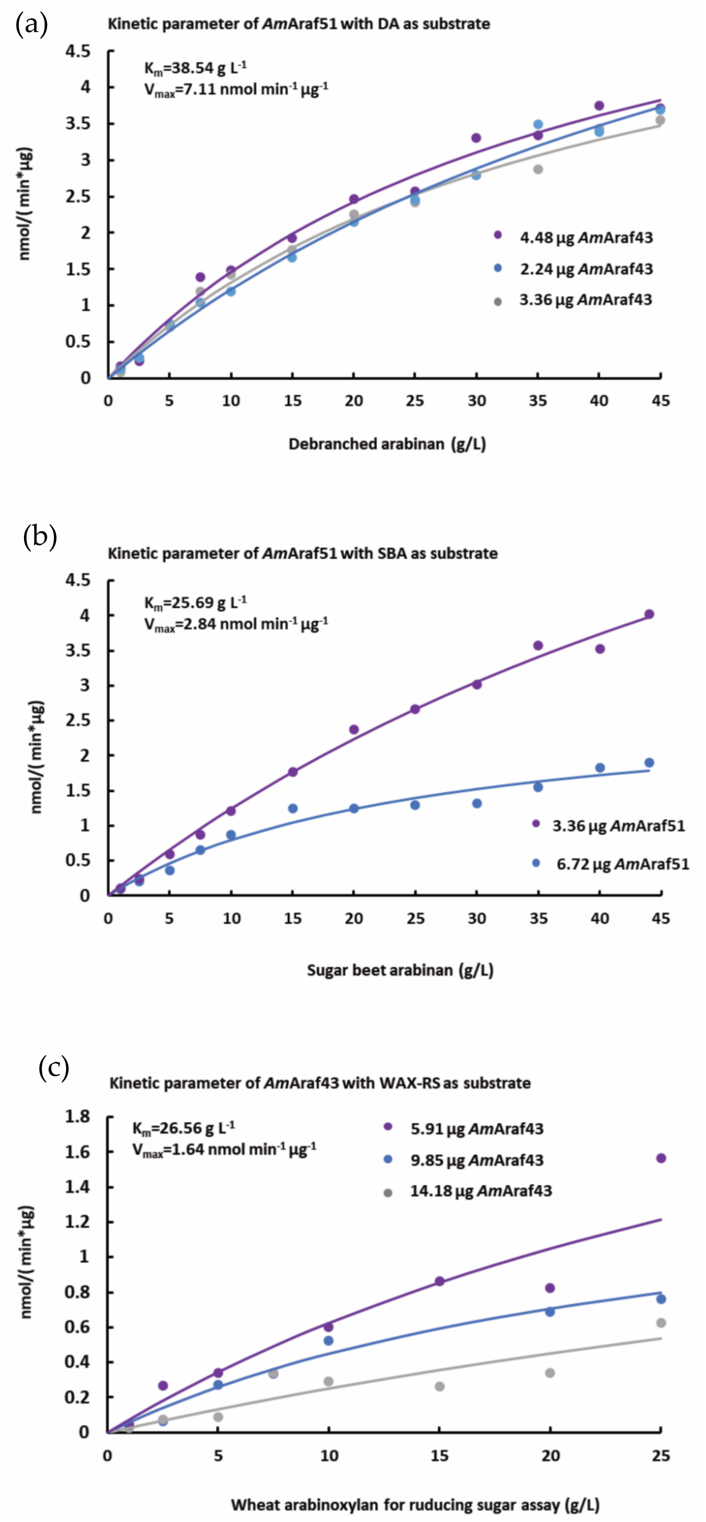

Supplementary Figure S2. Determination of kinetic parameters of (a) *Am*Araf51 degrading debranched arabinan, (b) sugar beet arabinan and (c) *Am*Araf43 degrading wheat flour arabinoxylan (for reducing sugar assay). Standard reactions (25 mM citrate phosphate buffer, at pH 6.0 and 60 °C for *Am*Araf51 and pH 5.0 and 50 °C for *Am*Araf43, 2 h incubation) with three different enzyme concentrations and different substrate concentrations were applied. K_m and V_{max} were determined by Microsoft Excel Solver. Error bars represent the standard deviation of duplicates.

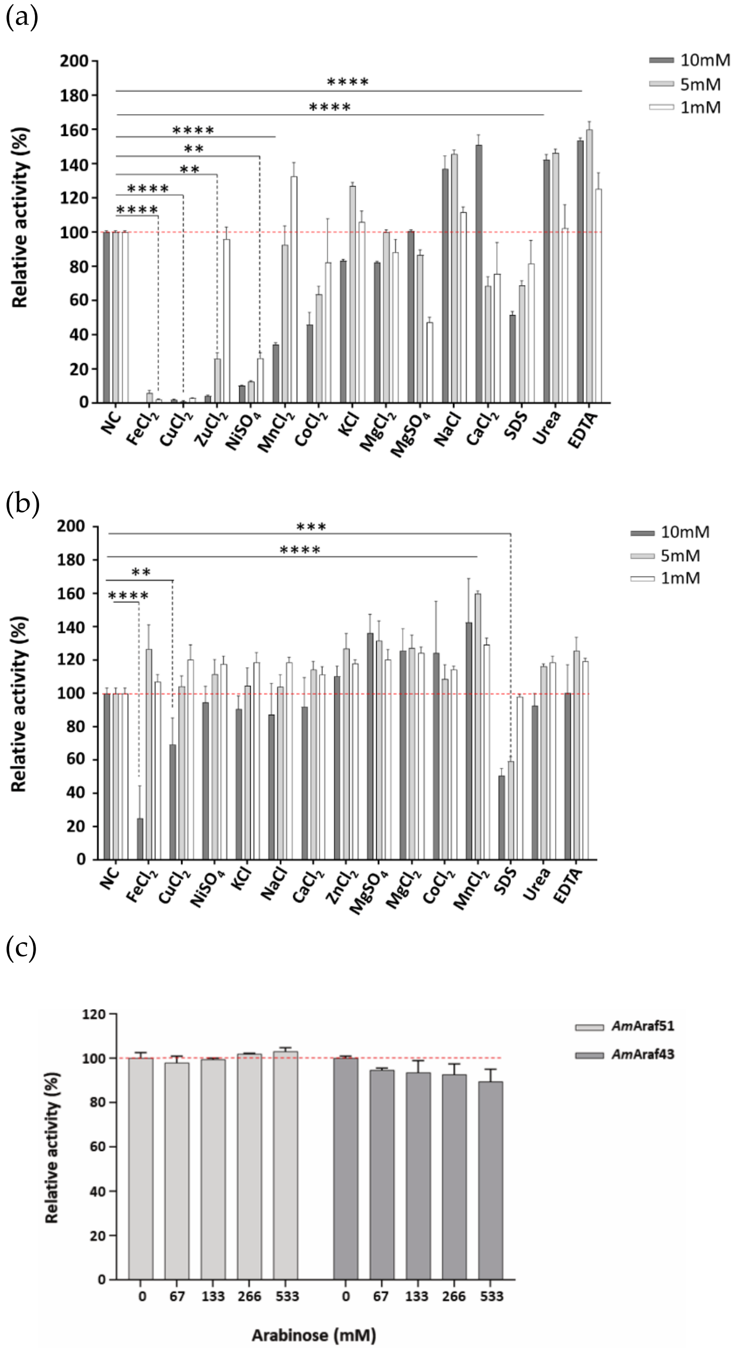

Supplementary Figure S3. Influence of metal ions, protein denaturants and metal chelator on the activity of (a) *Am*Araf51 and (b) *Am*Araf43. (c) Effects of arabinose on *Am*Araf51 and *Am*Araf43. Standard reaction (2 mM *p*NP-AF, 25 mM citrate phosphate buffer pH 6.0 and 4.5 for *Am*Araf51 and *Am*Araf43, respectively, 20 min) were performed as described in the materials and methods, using 0.56 µM *Am*Araf51 and 0.16 µM *Am*Araf43 with 1 mM, 5 mM and 10 mM salt solution, respectively, or with 67 mM, 133 mM, 266 mM, 533 mM arabinose, respectively. Activities are expressed relative to reactions without additional ions (H2O). Error bars show the standard deviation of triplicates.
